# Pathogenic Variants and Olipudase Alfa Treatment of Patients With Acid Sphingomyelinase Deficiency in Taiwan

**DOI:** 10.1002/mgg3.70204

**Published:** 2026-02-15

**Authors:** Hsu‐Heng Lin, Hui‐An Chen, Shyh‐Jer Lin, Rai‐Hseng Hsu, Ni‐Chung Lee, Wuh‐Liang Hwu, Yen‐Hsuan Ni, Yen‐Yin Chou, Pao‐Chin Chiu, Steven Shinn‐Forng Peng, Yin‐Hsiu Chien

**Affiliations:** ^1^ Department of Pediatrics National Taiwan University Hospital Taipei Taiwan; ^2^ Department of Pediatrics Changhua Christian Children's Hospital Changhua Taiwan; ^3^ Department of Medical Genetics National Taiwan University Hospital Taipei Taiwan; ^4^ Department of Pediatrics National Taiwan University College of Medicine Taipei Taiwan; ^5^ Division of Hematology/Oncology Veterans General Hospital‐Kaohsiung Kaohsiung Taiwan; ^6^ Center for Precision Medicine China Medical University Hospital Taichung Taiwan; ^7^ Hepatitis Research Center National Taiwan University Hospital Taipei Taiwan; ^8^ Department of Pediatrics, National Cheng Kung University Hospital, College of Medicine National Cheng Kung University Tainan Taiwan; ^9^ Department of Pediatrics Kaohsiung Medical University Gangshan Hospital Kaohsiung Taiwan; ^10^ Department of Radiology National Taiwan University Hospital and National Taiwan University College of Medicine Taipei Taiwan

**Keywords:** ASMD, NBS, Niemann–pick disease type A/B, Olipudase alfa, *SMPD1*

## Abstract

**Background:**

Acid sphingomyelinase deficiency (ASMD) is a rare lysosomal disorder with diverse clinical presentations and often delayed diagnosis. This study investigates the clinical features, genetic variants, and treatment outcomes in Taiwanese patients.

**Methods:**

We retrospectively reviewed nine ASMD cases in Taiwan, including genetic data and responses to olipudase alfa. Newborn screening data using the NeoLSD MS/MS kit for dried blood spot enzyme activity, followed by lyso‐sphingomyelin and molecular testing, were also analysed.

**Results:**

The *SMPD1* c.1497_1498inv variant was found in 62.5% of alleles among chronic neurovisceral ASMD cases, while c.995C > G appeared in 37.5% of chronic visceral ASMD cases and was also frequent in partial ASMD from newborn screening. Four patients received olipudase alfa; Patient 1, treated for 3 years starting at age 41, showed improved pulmonary function despite persistent thrombocytopenia and splenomegaly. Patients 2, 6, and 7, treated from early childhood, exhibited marked improvements in hepatosplenomegaly, interstitial lung disease, and growth within 1 year of therapy.

**Conclusion:**

This study highlights distinct genotype–phenotype correlations in ASMD and supports the clinical benefits of olipudase alfa. Increased awareness and early diagnosis, potentially through newborn screening, are essential for optimizing outcomes in ASMD.

## Graphical abstract

In Taiwan, the *SMPD1* c.1497_1498inv variant was common in chronic neurovisceral acid sphingomyelinase deficiency (ASMD), while c.995C > G was more frequently observed in chronic visceral ASMD. Four patients treated with olipudase alfa showed clinical improvements, particularly when treatment was started in early childhood.

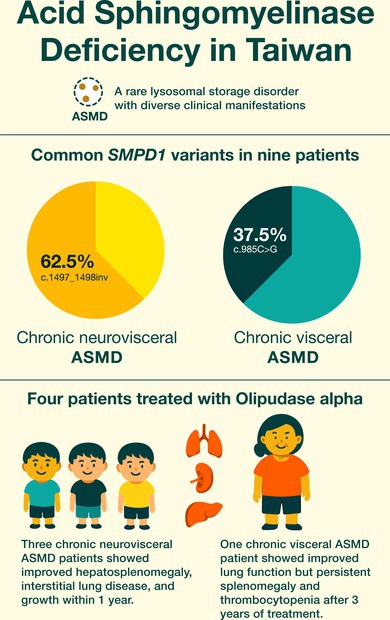

## 1 Introduction

Acid sphingomyelinase deficiency (ASMD), also known as Niemann–Pick disease types A and B, is an uncommon lysosomal storage disorder (LSD) caused by variants of the sphingomyelin phosphodiesterase 1 (*SMPD1*) gene (OMIM 257200, 607,616). These variants significantly reduce the activity of acid sphingomyelinase (ASM), a crucial enzyme responsible for sphingomyelin (SM) hydrolysis within lysosomes. Based on the degree of ASM deficiency and organs involved, ASMD can be classified as infantile neurovisceral ASMD (Niemann‐Pick disease type A), chronic neurovisceral ASMD (Niemann‐Pick disease type A/B), or chronic visceral ASMD (Niemann‐Pick disease type B) (Geberhiwot et al. 2023).

Olipudase alfa, a human recombinant enzyme, is a novel disease‐modifying enzyme replacement therapy (ERT) for ASMD that has been approved in the USA and several markets (Geberhiwot et al. 2023; Diaz et al. 2022) Clinical studies have demonstrated its efficacy in improving interstitial lung disease, liver enzymes, lipid profiles and hepatosplenomegaly (Diaz et al. 2022, 2021; Lachmann et al. 2023; Thurberg et al. 2020; Wasserstein et al. 2018), particularly in patients with chronic neurovisceral or visceral ASMD. Biochemical benefits eventually result in a better quality of life, including better exercise endurance, lower mortality, and improvements in growth parameters in children (Geberhiwot et al. 2023; Diaz et al. 2022; Lachmann et al. 2023; Wasserstein et al. 2018). Despite the therapeutic potential of olipudase alfa, the rarity of ASMD poses significant challenges in its diagnosis and management. Therefore, in this report, we summarize the genotypes and manifestations of nine patients with ASMD in Taiwan, four of whom received olipudase alfa treatment.

## 2 Materials and Methods

### 2.1 Ethical Compliance

This study was approved by the National Taiwan University Hospital and Kaohsiung Veterans General Hospital Institutional Review Board.

The National Taiwan University Hospital (NTUH), a tertiary medical center in Taiwan, contains a biochemical genetics lab service available to hospitals in Taiwan and nearby countries. We reviewed the clinical and laboratory data of Taiwanese patients who were diagnosed with ASMD at NTUH from 2000 to 2024. Leukocyte ASM activity was measured using 6‐Hexadecanoylamino‐4‐methylumbelliferylphosphorylcholine substrate (van Diggelen et al. 2005). Biomarker analysis was performed using tandem mass spectroscopy, as previously described (Polo et al. 2017, 2019). Genotyping was conducted using whole‐exome sequencing and confirmed using Sanger sequencing. Variants were annotated using NM_000543.5. Genomic coordinates were determined based on the GRCh38 reference genome and genomic reference sequence NC_000011.10. In addition, we conducted a literature review using the keywords “Niemann‐Pick” and “Taiwan” to identify previously published cases of ASMD in Taiwan.

The NTUH hosts a nationwide newborn screening (NBS) program that routinely screens 35%–37% of newborns in Taiwan. NBS for ASMD was added as a part of the LSD NBS in 2023. Dried blood spot (DBS) ASM activity was measured by the NeoLSD MSMS Kit (Revvity, USA) using a WATERS TQD UPLC–MS/MS system. DBS samples with ASM activity lower than the cutoff (1.45 μM/L/h, 30% of the normal mean) were subjected to lyso‐sphingomyelin (lyso‐SM) measurements and molecular analysis. A 2nd DBS was requested for confirmation (Polo et al. 2017, 2019). Newborns with persistent low DBS ASM activity, high lyso‐SM, and/or two pathogenic/likely pathogenic variants on the *SMPD1* gene were classified as screening positives and were requested to visit the NTUH for further management.

## 3 Results

### 3.1 Epidemiology and SMPD1 Variants in Patients

Five patients (patient 1–5) were diagnosed at the NTUH. Patient 1 was diagnosed at 29 years and treated with olipudase alfa at 41 years; patient 2 was diagnosed at the age of 2 years and treated at 2 years and 6 months; patient 3 was diagnosed with incidental splenomegaly during a CT scan for appendicitis at the age of 36 years; patient 4 developed hepatosplenomegaly at the age of 10 months and underwent bone marrow transplantation at the age of 17 months but died at the age of 13; patient 5 had experienced hepatosplenomegaly since infancy and developed neurodegeneration and died at the age of 2 years. Patients 6 and 7, previously reported by Pan et al., were diagnosed at the ages of 1.9 and 3.2 and were treated with olipudase alfa beginning at ages 2.5 and 5.7, respectively; (Pan et al. 2023) patient 8, previously reported by Lan et al., was diagnosed with hepatosplenomegaly at the age of 25 years; (Lan et al. 2009) and patient 9, reported by Lan et al., presented with splenic rupture at the age of 41 years (Lan et al. 2022). In total, one patient had infantile neurovisceral ASMD (patient 5), four had chronic neurovisceral ASMD (patients 2, 4, 6, and 7), and four had chronic visceral ASMD (patients 1, 3, 8, and 9) (Table 1).

**TABLE 1:** Overview of the genotype, phenotype, and treatment of nine Taiwanese patients with ASMD.

Patient	No. 1	No. 2	No. 3	No. 4	No. 5	No. 6	No. 7	No. 8	No. 9
**ASMD type**	Chronic visceral	Chronic neurovisceral	Chronic visceral	Chronic neurovisceral	Infantile neurovisceral	Chronic neurovisceral	Chronic neurovisceral	Chronic visceral	Chronic visceral
**Sex**	F	M	M	F	M	M	M	F	M
***SMPD1* variants**	p.(Tyr500His)	p.(Tyr500His)	p.(Cys223Gly)	# p.(Tyr500His)	# p.(Val513Ala)	p.(Tyr500His)	p.(Tyr500His)	# p.(Pro332Arg)	p.(Pro332Arg)
**Allele 1/Allele 2 (NM_000543.5)**	p.(His556Tyr)	p.(Arg498Pro)	p.(Pro332Arg)	# p.(Tyr500His)	# p.(Leu514ArgfsTer8)	c.1486 + 5G > C	c.1486 + 5G > C	# p.(Ala453Asp)	p.(Ala453Asp)
Age at onset (yr)	3	0.3	36	0.3	< 1	< 1	< 3	25	41
Age at diagnosis (yr)	29	2.5	36	1.2	2	1.9	3 .2	25	41
Initial presentation	Splenomegaly	Hepatosplenomegaly	Splenomegaly	Hepatosplenomegaly	Hepatosplenomegaly	NA	NA	Splenomegaly	Splenic rupture
**Clinical manifestation at diagnosis**									
Hepatomegaly	+	+	—	+	+	+	+	NA	—
Elevated liver enzyme levels	+	+	—	+	+	+	+	NA	NA
Splenomegaly	+	+	+	+	+	+	+	+	+
Thrombocytopenia	+	—	—	—	+	—	+	—	+
Anemia	—	—	—	—	+	NA	NA	—	+
Dyslipidemia	+	+	NA	+	+	+	+	NA	—
Developmental delay	—	+	—	+	+	+	+	NA	—
Abnormal brain MRI	NA	NA	NA	+	+	NA	NA	NA	NA
Interstitial lung disease	+	+	—	+	+	+	+	NA	—
Short stature	+ (< 3rd%)	+ (< 3rd%)	− (25 ~ 50th%)	− (25 ~ 50th%)	− (10th%)	+ (< 3rd%)	+ (< 3rd%)	NA	NA
Failure to thrive	+ (< 3rd%)	+ (25~50th to 3 ~ 10th%)	− (50 ~ 75th%)	− (10 ~ 25th%)	− (10th%)	− (3 ~ 10th%)	− (3 ~ 10th%)	NA	NA
**Biomarker and enzyme activity**									
Leukocyte ASM (nmol/mg protein/17 h, normal > 8.8 nmol/mg protein)	3.04	NA	NA	NA	2.87	NA	NA	2.56	NA
DBS ASM (μM/hr, normal > 1.45 μM/hr)	0.09	0.14	0.1	NA	NA	NA	NA	NA	0.15
Plasma Lyso‐SM (ng/mL, normal < 8 ng/mL)	305.9	872.5	58.31	NA	NA	351.9	635.7	NA	75.5
Treatment (age, yr)	ERT (41)	ERT (2.5)	Splenectomy (36)	BMT (1.5)	—	ERT (2.5)	ERT (5.7)	—	Splenectomy (41)
Prognosis	Improving abdominal distension, endurance	Improving abdominal distension, endurance	NA	Expired at 13 y/o	Expired at 2 y/o	Improving abdominal distension, endurance	Improving abdominal distension, endurance	NA	NA
Reference	This study					Polo et al. 2019		van Diggelen et al. 2005; Polo et al. 2017	

*Note:* The variants with # represent variant sequences transformed from the previous transcript to NM_000543.5 with two additional codons, without resequencing.

Abbreviations: +, present; −, absent; ASM, acid sphingomyelinase; BMT, bone marrow transplantation; DBS, dried blood spot; ERT, enzyme replacement therapy; F, female; M, male; MRI, magnetic resonance imaging; NA, not available; lyso‐SM, lyso‐sphingomyelin; y/o, years old.

The infant patient with neurovisceral ASMD (patient 5) had c.[1538 T > C];[1541delT] (p.[(Val513Ala)];[(Leu514ArgfsTer8)]) in the *SMPD1* gene; neither was seen in the other groups of patients. Among the four patients with chronic neurovisceral ASMD, all had the c.1497_1498inv (p.(Tyr500His)) variant on *SMPD1*, including one homozygous patient, making p.(Tyr500His) the most common variant (five of the eight alleles, 62.5%) in this group of patients. Among the four patients with chronic visceral ASMD, the c.995C > G (p.(Pro332Arg)) variant appeared in three patients, and the p.(Tyr500His) variant in one patient. Therefore, p.(Pro332Arg) was the most common allele (three of the eight alleles, 37.5%) in this group of patients.

### 3.2 p.(Pro332Arg) Variant Found by Newborn Screening

As of March 2024, the Newborn Screening Center at NTUH screened 59,123 newborns for ASMD. Twelve newborns exhibited slightly low ASM activity in their initial DBS samples (median 1.29 μM/L/h) (Table S1); however, all had normal ASM activity at the 2nd DBS and therefore were not patients. All but one had normal lyso‐SM levels (median 66.6, maximum 91.3, normal < 80 nmol/L). *SMPD1* gene sequencing revealed that 10 of the 12 newborns carried only one *SMPD1* pathogenic variant, with p.(Pro332Arg) representing six of the 10 alleles (60%). Two of the 10 newborns carried another c.1598C > T (p.(Pro533Leu)) variant, which was classified as a variant of unknown significance. The screening did not identify any patients with ASMD or individuals with homozygous or compound heterozygous pathogenic variants.

### 3.3 Treatment Outcomes

Four patients (patients 1, 2, 6, and 7) in Taiwan were treated with olipudase alfa according to the label instructions through a compassionate‐use program. Among the five patients who did not receive ERT, patient 3 had mild disease and was monitored, whereas patients 4 and 5 died and patients 8 and 9 were reported prior to the availability of olipudase alfa. After the dose‐escalation phase, the olipudase alfa dose was successfully titrated to 3 mg/kg in all patients. The expected treatment‐related adverse events, including infusion‐related allergic reactions, such as hypersensitivity and impaired liver function, were managed without complications. Specifically, one patient experienced mild, grade 1 infusion‐associated reactions (flushing and back pain), which were managed by slowing the infusion rate. Two patients had transient elevations in liver enzymes that resolved after slowing the initially escalating regimen. Another patient reported four episodes of post‐infusion febrile sensation, which were relieved with antipyretics.

As of December 2024, patient 1 had been receiving treatment for 3 years, patient 2 for 1 year, and patients 6 and 7 for 3.5 years (personal communication).

**FIGURE 1:**
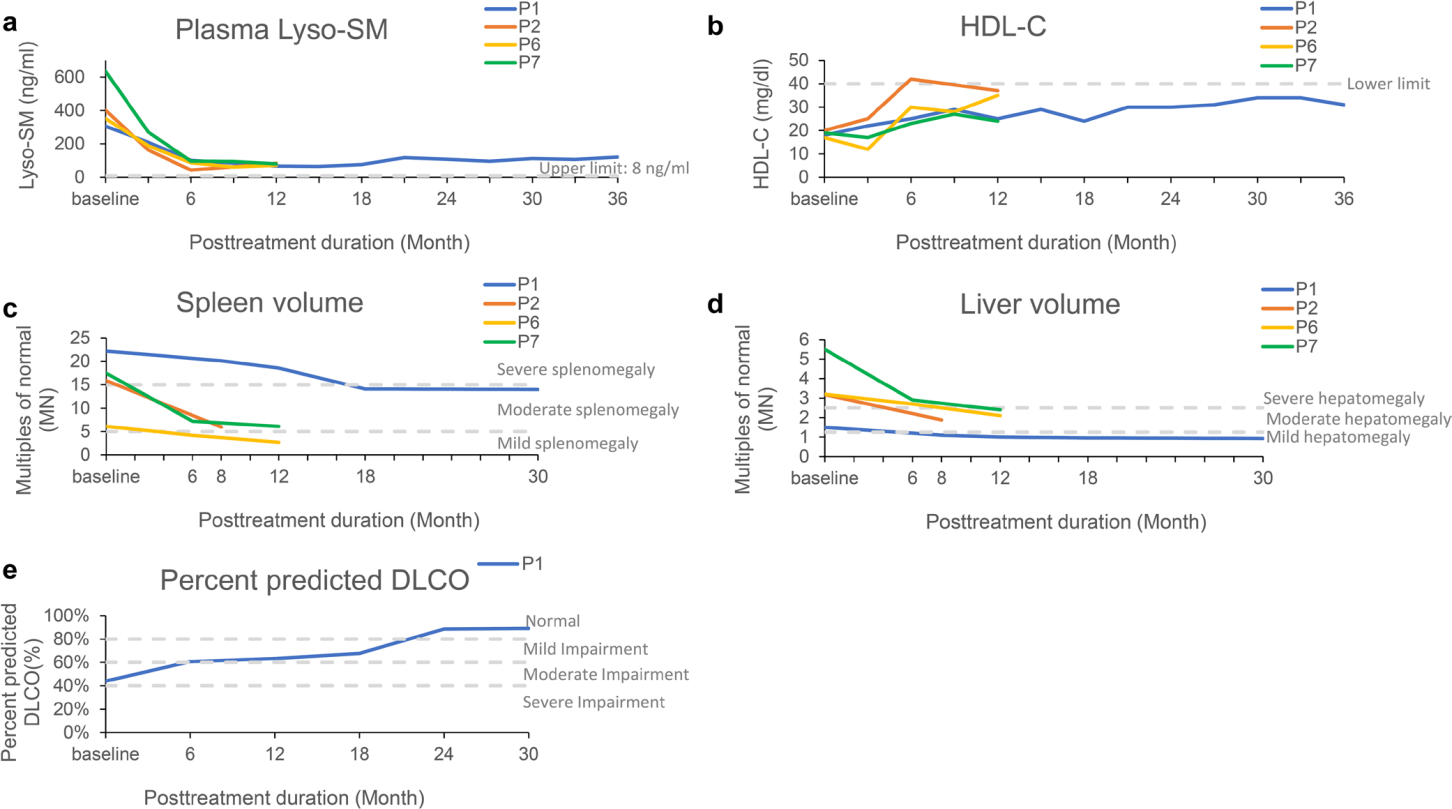
Changes in (a) plasma lyso‐SM levels, (b) HDL‐C levels, (c) spleen volume, (d) liver volume, and (e) lung diffusion capacity in four patients over time following treatment with olipudase alfa. Severe, moderate, and mild hepatomegaly are defined as > 2.5, > 1.25 to 2.5, and ≤ 1.25 multiples of normal (MN), respectively. Severe, moderate, and mild splenomegaly are defined as > 15, > 5 to ≤ 15, and ≤ 5 MN, respectively. DLCO was categorized as follows: No impairment, > 80%; mild impairment, > 60% to ≤ 80%; moderate impairment, > 40% to ≤ 60%; and severe impairment, < 40%. Abbreviations: DLCO: Diffusing capacity of the lung for carbon monoxide; HDL‐C, high‐density lipoprotein cholesterol; lyso‐SM, lysosphingomyelin.

Patient 1 presented with splenomegaly and thrombocytopenia at the age of 3. The diagnosis of ASMD was established at the age of 29 during workup to exclude Gaucher disease. Patient 1 began olipudase alfa treatment at the age of 41 with a short stature, severe splenomegaly, thrombocytopenia (platelet counts around 40 K/μL; normal range: 150–500 K/μL), interstitial lung disease visible on chest computed tomography (CT), impaired lung diffusion capacity, and exertional dyspnea. Additionally, the patient had a normal lipid profile, except for a low high‐density lipoprotein cholesterol (HDL‐C) level of 18 mg/dL (normal: > 50 mg/dL). After 36 months of therapy, the plasma lyso‐SM level of Patient 1 decreased from 305.9 ng/mL (normal < 8 ng/mL) to 107.4 ng/mL (Figure 1A), while the serum HDL‐C level gradually increased (Figure 1B). The diffusion capacity of the lungs for carbon monoxide (DLCO) improved from 44% to 89.1% (Figure 1E). However, splenomegaly (22.2 multiples of normal (MN)) showed only slight reductions to 14.0 MN (−37%) (Figure 1C), and thrombocytopenia remained the same.

Patient 2 presented with developmental delays and abdominal distension in the first year of life. At the age of 2, elevated liver enzyme levels were incidentally detected during admission for pneumonia. Hepatosplenomegaly and hyperlipidemia were also observed, including increased triglyceride (TG) levels of 311 mg/dL (normal < 150 mg/dL), total cholesterol levels of 286 mg/dL (normal < 200 mg/dL), low‐density lipoprotein cholesterol (LDL‐C) levels of 199 mg/dL (normal < 100 mg/dL), and HDL‐C levels of 20 mg/dL (normal > 40 mg/dL). Whole‐exome sequencing confirmed the diagnosis of ASMD at age 2 years and 6 months and a chest CT revealed interstitial lung disease. After 12 months of treatment, the plasma lyso‐SM levels of Patient 2 decreased from 402 ng/mL to 63 ng/mL (Figure 1A) and the AST, ALT, and lipid profiles returned to the normal ranges. The prominent hepatomegaly (3.2 MN) and splenomegaly (15.9 MN) gradually decreased to 1.9 MN (−40%) and 6 MN (−62%), respectively (Figure 1C,D). Follow‐up imaging revealed a reduction in ground‐glass opacity in the right upper lung.

According to the original report (Pan et al. 2023), patient 6 was diagnosed with chronic neurovisceral ASMD at the age of 1.9 years and treated with olipudase alfa at the age of 2.5 years, whereas patient 7 was diagnosed with chronic neurovisceral ASMD at the age of 3.2 years and treated with olipudase alfa at the age of 5.7 years. Both patients presented with hepatosplenomegaly, mild liver dysfunction, growth failure, interstitial lung disease, and developmental delays. During the first year of treatment, the patients showed improvements in liver and spleen size, liver stiffness (assessed by ultrasound elastography), growth metrics, lipid profiles, lyso‐SM levels, lung disease scores, and bone mineral density. These data were extracted from our previous original publication and incorporated into Figure 1 for comparison (Figure 1A–D).

## 4 Discussion

We collected data from nine patients with ASMD in Taiwan over 25 years. Considering the total number of births during this period, the incidence of ASMD was roughly 0.2 per 100,000. The estimated worldwide incidence of ASMD ranged from 0.4 to 0.6 cases per 100,000 (Kingma et al. 2015). Therefore, the diagnostic rate for ASMD in Taiwan may be less than 50%. The incidence of ASMD estimated from Taiwanese population genotyping data is 1.23 per 100,000 (Tsai et al. 2023) which is likely an overestimate.

The relationship between the genotype and phenotype in ASMD has been extensively discussed (Wasserstein and Schuchman 1993). Over 30% of ASMD cases have neurological manifestations (Wasserstein et al. 2006); however, the percentage varies according to the presence of founder variants in specific populations (McGovern, Avetisyan, et al. 2017). For example, neuropathic ASMD is more common in Ashkenazi Jewish people, and non‐neuropathic ASMD is predominant in North Africa (Wasserstein and Schuchman 1993). In the present study, the most common allele was p.(Tyr500His), which is associated with chronic neurovisceral ASMD. This variant has an allele frequency of 0.0003 (1/2984) in the Taiwan Biobank but was not observed in other populations. Hu et al. described 118 patients from Northern China, 64% of whom had non‐neuropathic ASMD (Hu et al. 2021). They found that the mean age at disease onset in patients homozygous for p.(Tyr500His) was 1.36 ± 0.59 years old, (Hu et al. 2021) which is compatible with the findings in the present study. The second most common allele in our patients, p.(Pro332Arg), was observed only in chronic visceral ASMD patients. This variant has an allele frequency of 0.006 (19/2984) in the Taiwan Biobank. Homozygous p.(Pro332Arg) (named as p.(Pro330Arg) in some studies) variant is also associated with milder forms of chronic visceral ASMD (Wasserstein et al. 2004). Therefore, p.(Pro332Arg) may possess sufficient residual ASM activity to prevent neurological symptoms.

To better understand the true incidence of ASMD in Taiwan, we screened 59,123 newborns for ASMD. Only 12 newborns with partial ASM deficiency were identified. Among them, 10 carried one pathogenic *SMPD1* variant, including six with p.(Pro332Arg), a variant also found in two of the 10 patients identified by the Illinois Newborn Screening Program. These two patients, both of whom were Asian ancestors, were diagnosed with chronic visceral ASMD (Hickey and Baker 2024a). The high incidence of p.(Pro332Arg) in newborn screenings was consistent with its high prevalence in the Taiwan Biobank. However, although the population prevalence of p.(Tyr500His) in the Taiwan Biobank was much lower than that of p.(Pro332Arg), p.(Tyr500His) was the most common allele found in our patients. This suggests that some individuals with either homozygous or compound heterozygous of p.(Pro332Arg) were not diagnosed. For example, patient 3 was incidentally found to have splenomegaly during a CT scan for appendicitis, whereas patient 9 presented with a life‐threatening splenic rupture. None of the patients had a previous suspicion of ASMD. Therefore, an abnormal lipid profile (low HDL‐C level, high LDL‐C level, or hypertriglyceridemia), unexplained liver dysfunction, or splenomegaly should trigger ASMD testing regardless of the patient‘s age (McGovern et al. 2004; Sen Sarma and Tripathi 2022). However, several issues would arise if we do pick up those adult onset, mildly affected patients by newborn screening shortly after birth. Defining a time or criteria to start ERT in those patients could be challenging, and the psychological impact during the long waiting period cannot be ignored (Gragnaniello et al. 2024; Hickey and Baker 2024b). Moreover, precise phenotype prediction after newborn screening, even though easier after the advancement in sequencing techniques, is not always possible (Wasserstein et al. 2019). Therefore, although newborn screening and early treatment of patients with early onset neurovisceral ASMD is very important, newborn screening for ASMD must be conducted cautiously.

Olipudase alfa demonstrated efficacy and tolerability in children and adults with ASMD (Diaz et al. 2022, 2021; Lachmann et al. 2023; Thurberg et al. 2020; Wasserstein et al. 2018). Patient 1 received olipudase alfa for 3 years with a good response in terms of lyso‐SM level, biochemistry, and lung‐diffusing capacity. However, splenomegaly and thrombocytopenia persisted. In Gaucher disease, thrombocytopenia typically improves only after the spleen volume decreases to below 1000 mL (Ha et al. 2017). Therefore, the limit in improvement of thrombocytopenia reflects a slow treatment response after long term sphingomyelin accumulation in the spleen. Prolonged sphingomyelin accumulation or dyslipidemia can also lead to irreversible effects, including short stature, cardiac valve disease, and early onset coronary artery disease (McGovern, Dionisi‐Vici, et al. 2017). Thus, despite an overall good treatment response to olipudase alfa, earlier intervention is still important and may have better efficacy.

## 5 Conclusion

ASMD in Taiwan has a specific epidemiological profile. The p.(Tyr500His) variant increases the incidence of neuropathic ASMD. The high prevalence of the p.(Pro332Arg) variant but its relatively lower representation in patients compared to p.(Tyr500His) in our cohort suggests an underdiagnosis of mild ASMD in Taiwan. With the availability of ERT and newborn screening, diagnosis and treatment of ASMD will soon enter a new era.

## Author Contributions

Conceptualization, Y.‐H.C, Y.‐H. N; formal analysis, H.‐H.L, S.‐J.L; investigation, P.‐C.C; data curation, Y.‐H.N, R.‐H.H, Y.‐Y.C, S.S.‐F.P, N.‐C.L; writing – original draft preparation, H.‐H.L; writing – review and editing, W.‐L.H, H.‐A.C, Y.‐H.C. All authors have read and agreed to the published version of the manuscript.

## Funding

The authors have nothing to report.

## Ethics Statement

This study was approved by the National Taiwan University Hospital Institutional Review Board (protocol code: 202403096RIND; date of approval: April 30, 2024) and Kaohsiung Veterans General Hospital Institutional Review Board (protocol code: 24‐CT9‐01(240813–3); date of approval: August 14, 2024), and was permitted to be published.

## Conflicts of Interest

The authors declare no conflicts of interest.

## Supporting information


**Table S1:** Results of newborn screening for ASMD. Dried blood spot (DBS) acid sphingomyelinase (ASM) activity units: μM/h, cutoff 1.45 μM/h; DBS lyso‐sphingomyelin (lyso‐SM) units: nmoL/L, normal < 80 nmoL/L. ^#^variant of unknown significance.

## Data Availability

The data that support the findings of this study are available from the corresponding author upon reasonable request.
