# Supplementary material for: Pathogenic Variants and Olipudase Alfa Treatment of Patients With Acid Sphingomyelinase Deficiency in Taiwan
Source: Mol Genet Genomic Med. 2026 Feb 15;14(2):e70204. doi: 10.1002/mgg3.70204 (PMC12906976; doi:10.1002/mgg3.70204)
Supplement: Supplementary file 1 — Table S1: Results of newborn screening for ASMD. Dried blood spot (DBS) acid sphingomyelinase (ASM) activity units: μM/h, cutoff 1.45 μM/h; DBS lyso‐sphingomyelin (lyso‐SM) units: nmoL/L, normal < 80 nmoL/L. #variant of unknown significance. [file MGG3-14-e70204-s001.docx]

**Pathogenic Variants and Olipudase alfa Treatment of patients with Acid Sphingomyelinase Deficiency in Taiwan**

**Hsu-Heng Lin^1,2^, Hui-An Chen^1,3,4^, Shyh-Jer Lin^5^, Rai-Hseng Hsu^1,3,4^, Ni-Chung Lee^1,3,4^, Wuh-Liang Hwu^1,3,4,6^, Yen-Hsuan Ni^1,4,7^, Yen-Yin Chou^8^, Pao-Chin Chiu^9^, Steven Shinn-Forng Peng^10^, Yin-Hsiu Chien^1,3,4^**

**Supplement**

**Table S1.** Results of newborn screening for ASMD. Dried blood spot (DBS) acid sphingomyelinase (ASM) activity units: µM/hr, cutoff 1.45 µM/hr; DBS lyso-sphingomyelin (lyso-SM) units: nmoL/L, normal < 80 nmoL/L. ^#^variant of unknown significance

| **No.** | **1^st^ DBS**  **ASM activity** | **2^nd^ DBS**  **ASM activity** | **1^st^ DBS**  **lyso-SM** | ***SMPD1* variant**  **Variant 1** | **Variant 2** |
| --- | --- | --- | --- | --- | --- |
| 1 | 1.35 | 2.28 | 67.46 | p.(Pro332Arg) | p.(Pro533Leu)^#^ |
| 2 | 1.22 | 1.64 | 65.15 | p.(Cys223Gly) | p.(Pro533Leu) ^#^ |
| 3 | 1.28 | 1.46 | 50.38 | p.(Pro332Arg) | - |
| 4 | 1.45 | 2.35 | 73.67 | p.(Tyr500His) | - |
| 5 | 1.34 | 1.80 | 91.30 | p.(Phe140Leu) | - |
| 6 | 1.21 | 2.70 | 62.47 | p.(Pro332Arg) | - |
| 7 | 1.30 | 2.48 | 76.69 | p.(Pro332Arg) | - |
| 8 | 1.15 | 3.20 | 42.81 | p.(Pro332Arg) | - |
| 9 | 1.39 | 3.30 | 64.76 | p.(Arg230His) | - |
| 10 | 1.24 | 1.67 | 75.15 | p.(Pro332Arg) | - |
| 11 | 1.24 | 2.67 | 33.58 | - | - |
| 12 | 1.44 | 5.03 | 33.46 | - | - |
